# User's guide to the orthopaedic literature: How to use an article about a randomized trial?

**DOI:** 10.4103/0019-5413.40246

**Published:** 2008

**Authors:** Brian Chan, Bernd Robioneck, Anders Joensson

**Affiliations:** Osteosynthesis and Trauma Care Foundation, Stryker Research and Development, Canada

**Keywords:** Clinical trials, critical appraisal, evidence-based medicine, hierarchy of evidence, randomized trials

## Abstract

Randomized trials, when well conducted, sit atop the hierachy of evidence. By limiting bias through randomization, concealment and blinding, surgeons can conduct high quality trials in orthopaedic surgery. The current article provides an easy, practical approach to critical appraisal of a randomized trial in orthopaedic surgery.

## CLINICAL SCENARIO

You are an orthopedic surgeon who is paged to the emergency department to evaluate and treat a seventy-one year old male complaining about osteoarthritic knee pain. He tells you that he is currently taking diclofenac as medication. He is overweight and exhibits symptoms such as swelling, stiffness and cracking of his right knee. There is also a significant reduction in the range of motion for his right knee. After carefully examining the patient's radiograph, you diagnose the patient with a severe case of medial tibiofemoral osteoarthritis.

There is clear indication that medications are not working for this patient and that surgery is warranted. Your initial thought is to perform an arthroscopy because it is the least invasive treatment. However, you recall reading a randomized control trial (RCT) regarding the inefficiency of arthroscopy for knee osteoarthritis. This makes arthroplastic surgery a much more attractive choice, due to the strong evidence supporting its effectiveness.

Captivated by this dilemma, you search the literature for articles on knee osteoarthritis and arthroscopy in order to decide which intervention to use, while your patient is taken to the operating room. The operating-room incharge nurse tells you that there are two other cases ahead of yours, which means that your case will be delayed for another 3 hours.

## THE LITERATURE SEARCH

In order to decide whether arthroscopy should be used or not, you queried online databases such as PubMed with the keywords: “knee” “osteoarthritis” and “arthroscopy.” In addition, you limited the results to randomized control trials only. This search yields only 37 articles, one of which is the article that you recall reading. The article is found in the New England Journal of Medicine and is titled “A controlled trial of arthroscopic surgery for osteoarthritis of the knee” by Moseley *et al.*^1^ This placebo-controlled study investigates the effectiveness of arthroscopic surgery for patients with osteoarthritis of the knee. Participants were randomly assigned to arthroscopic débridement, arthroscopic lavage or the placebo procedure (sham surgery). The study concluded that at no point did either of the arthroscopic groups report less pain or better function than the placebo group.

## THE GUIDE

Numerous historical cases have demonstrated the fallibility of intuition-derived conclusions. Evidence-based medicine encourages physicians to amalgamate their experiences and education with relevant research literature to provide proven-effective care.^2^ This guide embodies the values of evidence-based medicine and attempts to foster an understanding of the healthcare literature. Specifically, this piece exemplifies how one critically appraises such studies to evaluate their effectiveness as surgical therapies. As a clinician, this skill is vital because not all publications adhere to strict scientific standards, shedding skepticism on their conclusions. Thus, a clinician should be able to discriminate between a poor study and an excellent one. We suggest a three step approach when evaluating articles to guide patient care [Table 1].^3^

**Table 1:** User's guide to randomized trials in orthopaedics

**Validity**
Did experimental and control groups begin the study with a similar prognosis
Were patients randomized
Was randomization concealed
Were all patients analyzed in the groups to which they were randomized
Were patients in the treatment and control groups similar with respect to known prognostic factors
Did experimental and control groups retain a similar prognosis after the study started
**Blinding**
Did investigators avoid effects of patient awareness of allocation
Were patients blinded
Were aspects of care that affect prognosis similar in the two groups: were clinicians blinded
Was outcome assessed in a uniform way in experimental and control groups:
Were those assessing outcome blinded
Was follow-up complete
**Results**
How large was the treatment effect
How precise was the estimate of the treatment effect
**Applicability**
Can the results be applied to my patient
Were all clinically important outcomes considered
Are the likely treatment benefits worth the potential harm and costs

1. Can we believe the study?
2. Are the results large enough or precise enough?
3. Can they be applied to my patient?

### 1) Internal Validity | Can we believe the study?

When critiquing a study design and its subsequent findings, evaluation of the internal validity of the research paper is required. Internal validity is the ability of the study results to support a cause-effect relationship between the treatment and the observed outcome. In other words, the observed difference in outcome between groups is attributable only to the effect of the intervention under investigation. When assessing internal validity, there are six criteria to consider: adequacy of the allocation sequence; concealment of the allocation sequence; intention-to-treat (ITT) analysis; balance of prognostic factors, blinding; and completeness of follow-up. This section will examine each of the factors mentioned with a clinical perspective to assess the internal validity of the randomized placebo-controlled trial of knee arthroscopy by Moseley *et al.*

### Adequacy and Concealment of the Allocation Sequence | Did experimental and control group begin the study with a similar prognosis?

The importance of patient randomization can be seen in many drug trials that disprove initial promising results shown in observational studies. For instance, a randomized control trial (RCT) that evaluated the usage of a heart failure medication oral milrinone, showed an increase in morbidity and mortality of patients with this treatment despite beneficial results shown in prior non-randomized trials.^4^ In addition, other heart failure medication such as iboapmine and epoprostenol both showed positive results in observational studies, but when evaluated in the context of RCT trials, the results were contradictory, thus invalidating the prior non-randomized studies.^5^,^6^

Such contradictions are common between observational and RCT study designs. A safe heuristic is to trust RCTs over observational studies. They have been shown to be more reliable and less biased than observational studies.^7^–^10^ Hence, RCTs are considered to be higher in the hierarchy of research designs simply because they have the capability to randomize their patients to either the intervention or control group. Meanwhile, observational studies have minimal control over their groups. This additional degree of control minimizes the bias due to the adequacy of the randomized allocation sequence.

The adequacy of an allocation sequence is dependent on how well extraneous variables are controlled. It can always be implemented. A study has an adequate allocation sequence when researchers are unable to influence their patients' group assignments. With an inadequate allocation sequence however, researchers are able to influence which intervention their patients will receive. This will definitely bias the experiment and decrease the internal validity of the study.

In addition to controlling extraneous variables, the allocation sequence must be concealed from the researchers to avoid bias in the selection process. Otherwise, researchers may place patients with more severe symptoms in the treatment group out of sympathy, downsizing the treatment effect. Conversely, some researchers may do the opposite, upsizing the treatment effect.

A RCT that evaluated open versus laparoscopic appendectomy demonstrated a lack of allocation concealment because eligible patients at night were selectively chosen to undergo a specific procedure based on the preference of the attending surgeon.^3^,^11^ This was due to the attending surgeon's preference for open appendectomy surgery. He would deliberately hold translucent envelopes up to the light to dictate the allocation of surgery to be open appendectomy surgery. Thus if eligible patients are sicker at the night time, the surgeon's behaviour would create a systematic bias on the results against the open procedure.

Concealment can and should always be fully implemented to safeguard the allocation sequence before and until allocation. Trials where the allocation sequence concealment was compromised demonstrated (on average) a 30-40% overestimation of the actual treatment effect.^12^,^13^ When randomization is not concealed, the demographics of control and test groups are likely to become asymmetrical, introducing systematic errors in the measurement of treatment effects.^12^,^14^ This is called selection bias. A simple method that is quite popular in removing selection bias is the use of sealed, opaque, sequentially numbered and equally weighted envelopes, the contents of which allocate patients to a specific intervention. This precaution will preclude clinicians from selecting which intervention to give to a specific patient. Another method is to allocate patients from an independent central call-in centre. This means that each patient is registered and allocated to an intervention through an independent third party. In terms of rigor, the latter method is preferred because researchers are not involved at all in this process. However, this method tends to be harder to implement because of its intricacy and cost.

The article by Moseley *et al.*, stated that participants were randomly assigned to arthroscopic débridement, arthroscopic lavage or the placebo procedure. Furthermore, they used sealed, sequentially numbered and opaque envelopes to conceal the randomization sequence. Thus, both extraneous variables and selection bias are both well accounted for in this experiment.

### Intention to treat principle | Were all patients analyzed in the groups to which they were randomized?

Given the aforementioned importance of adequate allocation sequences and their concealment, maintaining the integrity of allocation sequences throughout the experiment is also pivotal. Thus, group allocations should also be final; regardless of whether patients received their prescribed regimen, they should be analyzed within their allocated group. This concept is known as the intention-to-treat (ITT) principle and serves several purposes. ITT analysis preserves the prognostic balance of groups that was achieved through randomization, fosters a greater sense of responsibility over patients' compliance, minimizes type I error (concluding a statistically significant difference when in fact there is none) and results in more generalizable, pragmatic results. A caveat to ITT analysis is that the magnitude of the treatment effect is typically decreased, increasing your chances of making a Type II error (finding no statistical difference when there is in fact a difference). However, there are exceptions to the ITT principle. Patients can be removed from the study post randomization if they do not have the disorder or when it is later determined that the patients are ineligible for participation in the study.^15^ In orthopedic trauma trials for example, participants must be enrolled and treated immediately after the traumatic incident. This makes diagnosis for comorbidities, which might have been part of the trial's exclusion criteria, impossible. Thus, it is typically acceptable to remove patients post randomization due ineligibility factors that are stated *a priori*. Ideally, an independent adjudication committee blinded to group allocation is responsible for this process - again safeguard against potential biases.

When evaluating the article of interest by Moseley *et al.*, there is no mention of patients not receiving their assigned intervention or an intention-to-treat analysis being performed. Therefore it is not possible to determine whether ITT analysis was conducted or not.

### Balanced prognostic factors | Were patients in the treatment and control groups similar with respect to known prognostic factors?

Maintaining a balance of prognostic factors between the treatment and control group affirms a direct association between the intervention and outcome as opposed to some extraneous factor. This will in turn lead to a better estimation of the treatment effect.

For instance, a trial evaluating corticosteroid as a treatment for osteoarthritis of the knee enrolls patients with low and high grade osteoarthritis. Patients diagnosed with high grade osteoarthritis have a much worse prognosis compared to patients with low grade osteoarthritis. Therefore in small scale randomized control studies with only limited number of patients in each intervention arm (ex. 10 patients), it is possible for a group to have the majority of high grade osteoarthritis. This will cause a serious bias in the study in favour of the other group mainly comprising of low grade osteoarthritis patients. However, if a trial were to enroll a lot more patients (ex. 1000), randomization would more likely achieve a balance of prognostic factors.

As depicted, there was a systematic error in the measurement of treatment effect caused by the measured variable's association with another causal factor (known as a confounder). This jeopardizes the internal validity of the study as a direct association between the intervention and outcome cannot be demonstrated. Experimenters almost always measure and compare the base-line characteristics of patients in each group to demonstrate similarities in prognostic and demographic variables.

Two commonly used methods to ensure balance of prognostic factors include stratification and blocking. These methods are also useful in ensuring equal group sizes. Stratification groups subjects based on known prognostic variables but doing so comes at the expense of reducing the unpredictability of the sequence. Therefore to counter this effect, blocking is often used in conjunction. Blocking ensures close balance of the numbers in each group at any time during the trial. For example, a block of six in a study with two groups will contain three patients in one group and three patients in another. The means by which the three are selected differ for each block (e.g. 1,2,5; or 4,5,6; or 1,4,6; etc.) Moreover, the size of these blocks varies, further adding to the unpredictability of the allocation sequence.

In the study performed by Moseley *et al.*, both stratification and blocking were used. Patients were stratified into three groups according to the severity of osteoarthritis (grade 1, 2 or 3; grade 4, 5 or 6; and grade 7 or 8). In addition, a stratified randomization process with fixed blocks of six was used. These two procedures resulted in a balance of prognostic factors for the study as indicated in Table 1 of the article. A total of 180 patients underwent randomization; sixty were assigned to the placebo group, sixty-one to the lavage group and fifty-nine to the débridement group; baseline characteristics were similar in all three study groups. This indicates that the Moseley *et al.* study demonstrates proper balancing of prognostic factors.

### Blinding | Did experimental and control groups retain a similar prognosis after the study started?

Blinding is the purposeful concealment of patients' group allocations to safeguard the randomization sequence after allocation. However it cannot always be implemented into every study because there are situations where personnel or study participants cannot be blinded for ethical or pragmatic reasons. It is important to distinguish blinding, which can't always be applied, from allocation concealment, which can always be done. Trials without blinding demonstrate, on average, a 17% overestimation of treatment effect.^16^ There are many ways to implement blinding; this user guide will examine some of them.

### Did investigators avoid effects of patient awareness of allocation: were patients blinded?

Blinding patients eliminates the psychological expectation of regimen effect, known as the placebo effect, which has a significant impact on the measured outcome. The best way of avoiding this is to keep patients unaware of whether they are assigned to the intervention or control.

Moseley *et al.*, simulated a standard arthoscopic débridement procedure in placebo patients and gave them identical postoperative care as patients in the arthoscopy arm. In addition to these procedures, the study assessed whether patients remained unaware of their treatment-group assignment through questionnaires. The patients were asked at each follow-up visit to guess which procedure they had undergone. Patients in the placebo group were no more likely than patients in the other two groups to guess that they had undergone a placebo procedure. For example, at two weeks, 13.8 percent of the patients in the placebo group guessed that they had undergone a placebo procedure and 13.2 percent of the patients in the lavage and débridement groups guessed that they had undergone a placebo procedure.

### Were aspects of care that affect prognosis similar in the two groups: were clinicians blinded?

Clinician blinding eliminates differences in patient care between the intervention and control group. This prevents the bias of mishandling cases that constitute exceptions to some generally expected conclusion and also preferential treatment of the intervention group.

In Moseley *et al.*'s study, it was not possible to blind the orthopedic surgeon. Despite this, the surgeon did not participate in any postoperative outcome assessments, thus ensuring the integrity of the study. Yet, it is unclear whether the surgeon participated in the postoperative care of the patients. Meaning clinician bias is possible, if patients were cared differently according to their treatment allocation.

### Was outcome assessed in a uniform way in experimental and control groups: were those assessing outcome blinded?

Blinding outcome assessors is especially important when the outcome is subjective such as determining the severity of osteoarthritis in the knee. In contrast, an objective outcome measure such as death does not require clinician blinding, because the outcome requires no interpretation. When outcome assessors are not blinded, interviewer bias occurs; this is the systematic error due to selective gathering of patient data, which could potentially bias outcome measures.^17^

The article by Moseley *et al.* showed blinding of outcome assessors because study personnel were unaware of the treatment-group assignments since the start of the study. Group assignments were only revealed to the operating surgeon, who did not participate in outcome assessment. Thus, outcome assessors were blinded throughout the study.

### Was follow-up complete?

During a clinical trial, investigators are interested in patients' outcome measure regardless of which group they were assigned to. Patients with unknown data are classified as lost to follow up. The greater the number of patients lost to follow up decreases the internal validity of a study. Data is rarely missing for trivial reasons; subjects that are missing typically have a different prognosis than those who remained in the study. Patients could have been lost to follow up because of an adverse outcome such as death or a very positive treatment outcome so that the patients did not return for further assessment. Incomplete follow-up biases the outcome measure.

Moseley *et al.*, demonstrated a moderate level of completeness to follow-up. Out of the 180 patients randomized, 164 completed the 24-month follow-up. Thus, nine percent were lost to follow-up. The validity of the study is only mildly compromised.

### Are the results of the study valid?

In general the study can be considered internally valid. All aspects of internal validity are in place except ITT analysis, which may compromise the integrity of the research paper. Please refer to Table 2 for the complete summary of internal validity seen in the Moseley *et al.*'s study.

**Table 2:** Validity assessment for the study regardiing arthoscopic surgery for osteoarthritis of the knee

Dates of report	2002
Were patients randomized?	Yes
Was randomization concealed?	Yes
Was an intention-to-treat analysis performed?	No
Were groups similar for baseline prognostic factors?	Yes
Were patients blinded?	Yes
Were surgeons blinded?	N/A
Were outcome assessors blinded?	Yes
Was follow-up complete?	Yes

## RESULTS

To estimate the size of the treatment effect, two questions have to be answered- 1) what is the single value most likely to represent the truth? and 2) what is the plausible range of values within which the true value may lie?

### How large was the treatment effect?

The size of the treatment effect refers to an estimate of the single value most likely to represent the truth to a study. Summary measures illustrate this value by measuring the central tendency along with measures of dispersion. In addition, outcome measures convey study results in various ways as listed in Table 3 of this paper.

**Table 3:** Measuring the treatment effect

**Summary measures**
Measures of central tendency
Mean, median, mode
Measures of dispersion
Standard deviation
Standard error
Variance
Range
**Outcome measures**
Incidence = the number of new events/the number exposed
The proportion of new events
Prevalence = the number of events/the number exposed
The proportion of events
Absolute risk (AR): Event rate in the control group
Absolute risk reduction (ARR) = AR control - AR treatment
The arithmetic difference in risk between two groups known as risk difference (RD)
Number needed to treat (NNT) = 1/ARR
The number of patients one would need to treat to prevent one event
Relative risk (RR) = AR treatment/AR control
The proportion of the original risk still remaining after therapy
Relative risk reduction (RRR) = ARR/AR control = 1 - RR
The proportion of the original risk (AR) removed by therapy
Hazard ratio (HR) = number of event / total observation time

**Figure 1:**
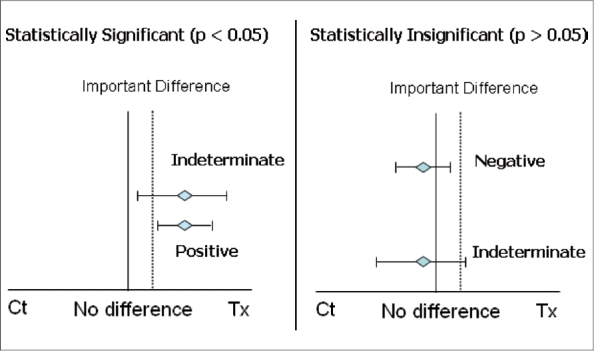
To identify whether a study is positive, indeterminate or negative, an understanding of statistical significance, minimally important difference and confidence interval of a trial is necessary. If outcome measures are statistically significant (refer to the graph on the left), then depending on whether the lower limit of the confidence interval passes through the minimally important difference line or not, the study can either be indeterminate or positive. Meanwhile, statistically insignificant results (refer to the graph on the right) will be either an indeterminate or negative study depending on whether the upper bound of the confidence interval passes through the minimally important difference line or not

In Moseley *et al.*'s randomized control trial, neither arthroscopic-intervention groups had greater pain relief than the placebo group (Figure 1, Table 2]. For instance, there was no difference in knee specific pain scale (KSPS) between the placebo group and the lavage group or the débridement group at one year (mean [±SD] KSPS scores, 48.9 ± 21.9, 54.8 ± 19.8 and 51.7 ± 22.4, (*P* = 0.14 for the comparison between placebo and lavage; *P* = 0.51 for the comparison between placebo and débridement) or at two years (mean KSPS scores, 51.6 ± 23.7, 53.7 ± 23.7 and 51.4 ± 23.2, respectively; P = 0.64 and P = 0.96, respectively). Similarly, there was no significant difference in arthritic pain between the placebo group and the lavage group or the débridement group at one or two years [Table 2]. Furthermore, at no time point did either arthroscopic-intervention group had significantly greater improvement in function than the placebo group (Table 3). Therefore, this study concluded that arthroscopic surgery for osteoarthritis of the knee had a minimal effect on the patient's pain relief, arthritic pain and improvement in knee function.

### How precise was the estimate of the treatment effect?

The estimate of the magnitude of a treatment effect is called a point estimate. However, it is unlikely that this estimate is precise. It is much more likely that the true effect lies between a range of values known as the confidence interval.^18^ Investigators arbitrarily set the confidence interval to 95%, meaning that if a study is repeated 100 times, the point estimate should lie within this interval 95 of the 100 times. It is interesting to note that the range of the 95% confidence interval is related to the sample size of the study. The larger the sample size, the larger the number of outcome events and the greater the precision of the study, which translates to a narrower confidence interval. Meanwhile statistical significance refers to study results and the probability that the observed difference between groups is due to chance. Statistical significance is represented by the p value and is conventionally set to 5%. If the p-value is less than the significance level, then the study results were not due to chance but an actual difference. However statistical significance does not tell you anything about the importance of the difference, if one was detected. For example, in study with a large enough sample size, even a small clinically unimportant difference will reach statistical significance. Minimally important difference (MID) is the smallest difference in score that is regarded as important and would persuade patients or clinician to consider a change in methods. MID can be determined by looking at the confidence interval and the value that exceeds the change expressed by the upper boundary of within-subject differences from clients who have not experienced an important change or the value that exceeds the change expressed by the lower boundary of within-subject differences from clients who have experienced an important change determined by any efficacy measure.

When study results show a statistically significant difference (*p* < 0.05), the confidence interval and MID can determine whether the study is a positive study or an indeterminate study. If the confidence interval crosses MID then it would be an indeterminate study; meanwhile if it is above and not overlapping of MID, then it would be a positive study. On the other hand, if the trial results show a statistically insignificant difference (*p* > 0.05), a study would be concluded indeterminate when the upper limit of the confidence interval overlapped with MID. Conversely, a statistically insignificant difference with confidence intervals completely below MID is considered a negative trial.^19^ Please refer to [Figure 1] for an illustration of the concept mentioned above.

The RCT conducted by Moseley *et al.*, is lacking evidence proving the superiority of the arthroscopic treatments over the placebo procedure in relieving pain or improving function (*p* > 0.05). The 95% confidence intervals for the differences in outcome between each arthroscopic procedure and the placebo procedure excluded important differences. The minimally important differences used for this evaluation were as follows: a difference of 13.5 points on the KSPS, 10.0 on the AIMS2-P, 11.8 on the SF-36-P, 12.8 on the AIMS2-WB, 11.3 on the SF-36-PF and 4.5 on the PFS. At almost all time points during follow-up (72 of 84 comparisons), the confidence intervals could not surpass MID. Therefore the study can be concluded as a negative trial.

### Applicability/Generalizability

#### Can the results be applied to my patient?

Regardless of how valid or how clinically significant results from a study are, suitability to the patient's situation is integral for practicing clinicians. When appraising a study, it is critical to determine the research question. A useful acronym is PICOT, which stands for patient, intervention, control, outcome of interest and timeframe. For example, a research question can be “does meniscal repair using inside-out suturing lead to reduced rates of failure of the repair compared to an all-inside technique in patients with longitudinal tears of the meniscus in a 24 months postoperative follow-up study?” In addition to comparing the characteristics of study participants and your clinical patients, a determination of whether the trial was explanatory (conducted in ideal situation) or pragmatic (conducted in typical situation) is necessary. Explanatory studies question the intervention work under “ideal” circumstances when applied by expert clinicians to study participants who are at high risk of a bad outcome, are highly responsive to the intervention and are highly compliant. Meanwhile, pragmatic trials assess whether the intervention work under “usual” circumstances when offered to all comers with the condition of interest. Please note that each study lies within a continuum between explanatory and pragmatic, attributing to various factors as outlined in Table 4. Further applicability can be determined by looking at the inclusion and exclusion criteria and additional subgroup analyses of a study.

**Table 4:** Applicability of study results

Attribute	Explanatory	Pragmatic
Eligibility criteria	Very strict, limited to high-risk, highly responsive, highly compliant participants	All comers
Post-hoc exclusion	Blinded adjudication of all includes; exclusion of ineligibles from the analysis	Includes all randomized patients
Intervention	Given by the best hands and closely monitored for dose and side-effects	As in routine clinical care
Intensity of follow-up	High, with frequent visits	No greater frequency than in routine practice
Duration of follow-up	Follow-up stops as soon as patient stops complying with treatment	Follow-up continues until death or the end of the trial
Participant compliance	Closely monitored, compliance strategies in place	Monitored unobtrusively if possible; no strategies in place
Clinician adherence to the study protocol	Closely monitored, with feedback for incomplete performance	Little or no monitoring
Events of interest	Primary analysis restricted to events that answer the biological/educational/organizational question or constitute adverse effects of the intervention	Primary analysis includes all events, good and bad, regardless of their causes

#### Are the likely treatment benefits worth the potential harm and costs?

Treatment benefits are desired when internally valid studies show significant treatment effects that are applicable to your patients. However, a clinician should not only consider the benefits and risks associated with the intervention but also whether the benefits are worth the healthcare resources expended on them. In the RCT conducted by Moseley *et al.*, the efficacy of arthroscopic lavage or débridement in patients with osteoarthritis of the knee is no greater than that of placebo surgery. Therefore the implications are that the billions of dollars spent on such procedures annually might be put to better use. However, care should be taken in the interpretation of the results as this is a pilot study.

## RESOLUTION OF THE SCENARIO

In conclusion, once the orthopedic surgeon has found an article of interest on surgical therapy, it is vital for them to assess the internal validity of the research paper using the principles presented in the user guide. Trials with strong internal validity will reinforce the inferences made and support clinical decisions. In addition, one must determine whether the magnitude and precision of the results qualify the study to be positive, indeterminate or negative. Afterwards, the generalizability of a study to a patient in the clinical setting is absolutely necessary since the results are sensitive to the medical situation of the patient. Lastly, it is important to compare the relative benefits and risk of the intervention.

Given the opening vignette and our appraisal of Moseley *et al.*'s study, arthoscopic surgery for osteoarthritis of the knee does not seem like an effective treatment in terms of pain relief, arthritis pain and improvement in knee function. Therefore, the clinician should consider arthroplastic surgery as an alternative treatment, which has been proven to be more effective as outlined in the opening scenario.

The purpose behind this entire article is to exemplify the critical reading process used when analyzing research literature. This is an important skill to develop as physicians often base their clinical decision on scientific research. This guide promotes evidence-based medicine and attempts to foster an understanding of the healthcare literature as an essential component of clinical decisions.

To identify whether a study is positive, indeterminate or negative, an understanding of statistical significance, minimally important difference and confidence interval of a trial is necessary. If outcome measures are statistically significant (refer to the graph on the left), then depending on whether the lower limit of the confidence interval passes through the minimally important difference line or not, the study can either be indeterminate or positive. Meanwhile, statistically insignificant results (refer to the graph on the right) will be either an indeterminate or negative study depending on whether the upper bound of the confidence interval passes through the minimally important difference line or not.
